# Improving Support Through Technology: Pilot Testing of a User-Centered Mobile Health App for Family Caregivers

**DOI:** 10.1093/geroni/igaf122.925

**Published:** 2025-12-31

**Authors:** Haomin Hu, I Made Agus Setiawan, Zara Ambadar, Andi Saptono, Heidi Donovan, Brad Dicianno, Bambang Parmanto, Yong Kyung Choi

**Affiliations:** University of Pittsburgh, Pittsburgh, Pennsylvania, United States; University of Pittsburgh Department of Health Information Management, School of Health and Rehabilitation Sciences, Pittsburgh, Pennsylvania, United States; University of Pittsburgh Department of Physical Medicine and Rehabilitation, School of Medicine, Pittsburgh, Pennsylvania, United States; University of Pittsburgh Department of Health Information Management, School of Health and Rehabilitation Sciences, Pittsburgh, Pennsylvania, United States; University of Pittsburgh, Pittsburgh, Pennsylvania, United States; University of Pittsburgh Department of Physical Medicine and Rehabilitation, School of Medicine, Pittsburgh, Pennsylvania, United States; University of Pittsburgh Department of Health Information Management, School of Health and Rehabilitation Sciences, Pittsburgh, Pennsylvania, United States; University of Pittsburgh, Pittsburgh, Pennsylvania, United States

## Abstract

Family caregivers (caregivers) often begin their caregiving roles unexpectedly without adequate training or support. They prioritize caregiving over self-care, leading to compromised health outcomes. In response, we employed user-centered approaches to interview caregivers, co-design solutions, and develop a multi-component Family Caregiver App. While many digital solutions focus solely on caregiving skills, our Family Caregiver App uniquely integrates self-care alongside caregiving education. This study aimed to evaluate the app’s usability, acceptability, and feasibility in a three-week cohort with 12 caregivers averaging 44.5 (±13.69) years of age. Usability was measured with the Mobile Health App Usability Questionnaire (MAUQ), ranging from 1 (disagree) to 7 (agree), resulting in an average score of 5.49 (±0.92). Acceptability, assessed through MAUQ subscales (ease of use: 5.62 ± 0.72, usefulness: 5.11 ± 1.35) and qualitative feedback guided by the Technology Acceptance Model (TAM), revealed positive user perceptions. Caregivers highlighted the app’s simple and accessible user interface, intuitive prompts, and straightforward navigation as key facilitators of ease of use. Additionally, cross-platform compatibility and tailored caregiver-focused features contributed significantly to the perceived usefulness and participants’ intentions for continued app use. Feasibility metrics indicated high engagement, with all caregivers completing the study and meeting predefined app usage criteria (average usage >1/3 days across more than four modules). Our findings consistently demonstrate that the Family Caregiver App is usable, acceptable, and feasible. Integrating user-centered and inclusive design principles offers a promising avenue to support caregivers, holistically addressing their caregiving responsibilities and well-being.

